# Adaptation and pilot implementation of a hereditary cancer risk-assessment tool for primary care

**DOI:** 10.1186/s12875-025-02935-6

**Published:** 2025-08-06

**Authors:** Sukh Makhnoon, Anoop Gurram, Eyad Alrabbat, Tiwatope Ibidapo, Ying Ma, Emanuel Villa, Michael E. Bowen, Sayoni Lahiri, Celette Sugg Skinner, Sara Pirzadeh-Miller, Steven Leach

**Affiliations:** 1 Peter O’Donnell School of Public Health, University of Texas Southwestern Medical Center, 5323 Harry Hines Blvd, Dallas, TX 75390 USA; 2 Simmons Comprehensive Cancer Center, University of Texas Southwestern Medical Center, Dallas, TX USA; 3 Medical School, University of Texas Southwestern Medical Center, Dallas, TX USA; 4 Cancer Genetics Program, Simmons Comprehensive Cancer Center, University of Texas Southwestern Medical Center, Dallas, TX USA; 5 Division of General Internal Medicine, University of Texas Southwestern, Dallas, TX USA

**Keywords:** Hereditary cancer, Risk assessment, Adaptation, Implementation

## Abstract

**Background:**

Family history-based risk assessment for hereditary breast and ovarian cancer is guideline-recommended but clinical implementation remains limited. This is likely, in part, because it adds to the limited time primary care providers (PCPs) have to implement all guideline-recommended care.

**Methods:**

We adapted Family History Screening 7 (or FHS7), designed for administration by a PCP, for self-report by primary care patients. We used the Framework for Reporting Adaptation and Modifications to Evidence-based Implementation Strategies (FRAME) to guide the modifications. We conducted a pilot feasibility study of hereditary prevention program using the adapted risk-assessment tool and report results from the first year of the program (February 2023-March 2024).

**Results:**

Feedback from clinical stakeholders and our literature review revealed that, while hereditary cancer risk assessment was a priority for the primary care setting, implementation by PCPs was not feasible. We therefore adapted FHS7 for patient self-report by separating double-barreled items and eliminating jargon, resulting in nine items– six with binary (yes/no) and three with numeric responses. Outcomes from pilot implementation of the adapted FHS7 (n=4,355) showed high completion rate (77% completed all items), with greater completion via MyChart than in-person (87% vs. 13%), and higher non-response for the three items with numeric responses compared to the six with binary responses. Overall, positivity rate of the adapted FHS7 was 36%.

**Conclusion:**

This paper describes our team’s process of adapting the FHS7 questionnaire to retain the core function (evaluating specific family history of cancer information) while adapting to fit the clinical context. Preliminary implementation data suggest high completion rate in the primary care setting.

## Background

Family history-based risk assessment for hereditary breast and ovarian cancer (HBOC) is guideline-recommended and has strong evidence supporting implementation into clinical practice [1]. Risk assessment is conducted by evaluating an individual’s family history of cancer, with follow-up genetic counseling and/or testing for those deemed to be at increased risk. The United States Preventive Services Task Force (USPSTF) [2] has recommended risk assessment for HBOC in primary care since 2013 as a grade B recommendation and has identified tools to support risk assessment. Despite their proven efficacy [3], these tools often require administration by time-constrained primary care providers (PCP) and are therefore not always well-suited for implementation in real-world clinical settings.

Implementation of guideline-indicated HBOC screening in primary care is challenging due to competing demands faced by PCPs to both manage acute and chronic disease and provide guideline-recommended care [4]. Estimates suggest that PCPs would require 27 h per day to provide all guideline-recommended preventive care [4]. Requiring PCPs to administer a family history-based questionnaire exacerbates this time scarcity and likely contributes to the implementation gap. Studies show that only 33–40% primary care providers collect detailed cancer family history information [5–8], in part due to time constraints in gathering detailed history during primary care appointments [9], limited awareness of genetic evaluation guidelines [10], and limited resources for follow-up genetic counseling and testing [11]. Addressing this implementation challenge can help streamline hereditary cancer risk assessment. In this study, we modified a PCP-facing risk assessment tool to self-reported patient-facing format and leveraged the electronic health record (EHR) system to automate risk-determination and further facilitate implementation.

The Family History Screen 7 or FHS7 is an USPSTF-endorsed, evidence-based tool that identifies individuals at high-risk for HBOC. Comprised of seven items, its short length makes it an excellent fit for most clinical settings [13]. However, a key limitation to implementation is the required PCP involvement. Family history information derived from EHR alone is inadequate to complete FHS7; it is known to severely underestimate the prevalence of FHS7 positive patients [14]. Therefore, we adapted FHS7 to fit better within primary care. Adaptations to existing evidence-based interventions are often necessary to improve their fit within the delivery setting and documenting and reporting results from such adaptations are essential to advance implementation research [15]. Here, we report on the adaption of FHS7, a hereditary cancer risk assessment tool, and outcomes from its pilot implementation within a primary care setting. We used the Framework for Reporting Adaptations and Modifications to Evidence-based Interventions (FRAME) [16] to characterize the adaptation and report results. FRAME provides a systematic approach to report adaptations using these characteristics: (a) when and how modifications were made, (b) whether the modification was planned or reactive, (c) who determined the modifications, (d) what is modified, (e) how delivery is impacted, (f) type of modifications to context or content, (g) impact on fidelity, and (h) reasons for the modification, including the intent, goal, and context.

## Methods

The adapted risk assessment tool was piloted as part of a multistage hereditary cancer risk-assessment program. The program involved sequential use of several evidence-based interventions: (1) risk assessment through family history evaluation (2), patient navigation (3), genetic counseling for at-risk individuals (4), genetic testing if recommended, and (5) follow-up care coordination for carriers of pathogenic variants. Here, we describe the first step of the hereditary cancer risk assessment program– adaptation and pilot implementation of the risk assessment tool, FHS7.

### Setting

This program was implemented at the University of Texas Southwestern Medical Center’s Multi-Specialty primary care clinic. The Multi-Specialty Clinic provides comprehensive primary care, including preventive health services and management of chronic diseases, and serves adults in the Dallas Forth Worth Metroplex. Following adaptation in early 2023, the adapted FHS7 was implemented at the Multispecialty clinic in February 2023.

### When the modification was made

At the outset, we identified the lack of family history-based risk assessment as the problem at this primary clinic. Our needs assessment was informed by: (1) low rate of genetic counseling referrals from this primary care setting, (2) known barriers to family history-based risk assessment in primary care settings, (3) burden of hereditary cancers in the geographic area, and (4) engagement with the clinic leader (SL). We found that, although family history of cancer was documented in the family history module of EpicCare– the EHR module used at the institution and populated either by the patient via MyChart or clinical staff or providers during routine office visits– data capture was inconsistent and lacked sufficient detail to assess hereditary cancer risk [17, 18]. Therefore, completion of hereditary cancer risk assessment was identified as the overall outcome to be achieved prior to implementation.

### What was modified and reason for modification

We identified six candidate evidence-based tools specified by the USPSTF [1] and selected the 7-question family history screening tool [3, 19] or FHS7. FHS7 is a validated instrument for familial risk assessment that has been previously tested in community settings [3].

Similar to prior literature [13], we selected FHS7 because it was least complex, short in length, and required minimal scoring, thus making it a good match with the priority population and an excellent fit for our organizational context. However, because FHS-7 was designed to be administered by a PCP rather than via self report, FHS7 required adaptation to fit with the specific needs of our implementation setting. Although the form of administration of FHS7 was adapted from being PCP-reported to patient-reported, its function and core were retained. Modifications are summarized in Table 1 and described in the results section.

**Table 1:** Adaptation of the 7-item family history screening (FHS7) from provider report to patient report

Original FHS7	Adapted FHS7
Did any of your first-degree relatives have breast or ovarian cancer?	1. Did any of your first-degree relatives (parent, children, siblings) have *breast* cancer? (yes/no)
	2. Did any of your first-degree relatives (parent, children, siblings) have *ovarian* cancer? (yes/no)
Did any of your relatives have bilateral breast cancer?	3. Did any of your relatives have *bilateral breast* cancer (i.e., cancer in both breasts)? (yes/no)
Did any man in your family have breast cancer?	4. Did any man in your family have *breast* cancer? (yes/no)
Did any woman in your family have breast and ovarian cancer?	5. Did any woman in your family have both *breast and ovarian* cancer? (yes/no)
Did any woman in your family have breast cancer before age 50y?	6. Did any woman in your family have *breast* cancer before the age of 50 years? (yes/no)
Do you have 2 or more relatives with breast and/or ovarian cancer?	7. How many relatives with *breast* cancer are in your family? ____
Do you have 2 or more relatives with breast and/or bowl cancer?	8. How many relatives with *bowel* cancer (colon/rectal) are in your family? _____
	9. How many relatives with *ovarian* cancer are in your family? _____

Wording on parts of question 1,2,3,5,7,8 & 9 were altered to improve comprehension

### Pilot implementation of adapted FHS7

For pilot implementation and evaluation, we developed additional educational materials for the primary care clinic, hired a genetic patient navigator to facilitate follow-up, developed a navigation tracking and outcomes database, and collaborated with a clinical champion (SL) and EPIC application analyst (EV) to implement the adapted FHS7 in the primary care clinic. Two weeks before a scheduled annual primary care appointment, each patient receives a message in the patient portal prompting them to complete the adapted FHS7 alongside other intake materials. Patients who do not complete it prior to their appointments have the option to do so with the intake nurse during the appointment. One positive response in the original 7-item questionnaire initiates referral to genetic counseling.

### Fidelity of adaptation

Because pre-adaptation outcome data were not possible to obtain at this clinic, we examined theoretical fidelity, rather than empirical fidelity, to assess the degree to which the adapted intervention was delivered as intended (i.e., fidelity). The adapted instrument stayed true to the function of the original version, by measuring the same family history of cancer and using the same algorithm to determine high-risk patients.

### Data collection

We collected data from the electronic data warehouse to understand characteristics of patients who respond to the questionnaire. Working with information resource analysist (YM), we collected EHR data on how (electronic patient portal or in-person), which (new vs. returning patients, sociodemographic characteristics), and when (date, time of day) patients completed adapted FHS7. For patients who also underwent genetic counseling (GC), a research assistant completed the adapted questionnaire items using manually extracted family history data recorded in patients’ pedigrees by the genetic counselor. GC-collected information is considered the gold standard for family history data whereas PCP-collected family history data, although more contextually relevant, is not routinely collected in this practice setting. The research assistant was blinded to patients’ self-reported family history information.

#### Statistical analysis

We report descriptive characteristics using means and medians and comparisons of categorical variables performed by Chi-squared test or Z-test for proportions. For each high-risk patient who completed a follow-up GC visit, we compared the self-reported and GC-collected family history data to compute point estimates (Kappa and % agreement) and 95% confidence intervals for concordance. We ran separate multivariable regression models to compare associations between item completion (total, binary survey items [yes/no], and numeric survey items), patient demographics (age, sex, race, ethnicity), patient characteristics (new or established patient), and questionnaire response method (MyChart or at-clinic). In multiple regression, the race categories American Indian or Alaska Native and Native Hawaiian or Other Pacific Islander were combined into ‘other’ due to small sample sizes. All statistical analyses were performed in SAS.

## Results

### The modifications

Key adaptations to FHS7 included separating double-barreled items into separate questions and explaining jargon such as ‘bilateral’ to increase patient comprehension. In doing so, the original seven item questionnaire was expanded to nine items (Table 1). MyChart was selected as the primary modality for administering the questionnaire, given high adoption rate of the electronic patient portal at this primary care clinic. Those who did not complete the questionnaire via MyChart had the questionnaire administered by an intake medical assistant during the in-person appointment.

### Who determined the modification

We relied on literature review, stakeholder feedback, and professional expertise to adapt the FHS7 from its original administration mode of interview via a PCP to our administration mode of patient self-report (Table 2).

**Table 2:** Summary of adaptations for FHS7 according to the framework for reporting adaptations and modifications to Evidence-based interventions (FRAME)

Domain	Adaptations
What areas/aspects were modified	Content
	Evaluation
	Implementation
Who participated in recommending and deciding the modification	Research team
	Administrator
	Service provider
When the modification occurred	Pre-implementation
Whether the modification was planned	Planned and proactive adaptation
Whether the modification was fidelity consistent	Fidelity-consistent
Whether the modification was a temporary drift	Broad change for the extended period
At what level of delivery the modification was made	Entire target group
Why the modification was made (i.e., reasons, goals, rationales)	To improve fit with recipients
	To increase engagement
	To reduce cost

### How delivery is impacted

All patients seen at the clinic were invited to report their family history of cancer using the adapted FHS7. From February 2023 to March 2024, 4,355 primary care patients were invited to complete the FHS7; 3,338 (77%) completed all nine items, 567 (13%) completed it partially, and 450 (10.3%) did not respond to any items. More patients responded to the questionnaire via MyChart than at the clinic (87% vs. 13%) but item completion rates were higher for in-person than MyChart (90% vs. 75%). Completion rates were similar between new and established patients at the clinic (79% and 76%, respectively). Patient demographics are shown in Table 3. Most questionnaires were completed between 6am and 6pm. Overall, positivity rate of the adapted FHS7 was 36%.

**Table 3:** Sociodemographic characteristics of primary care patients (*N* = 4,355)

Characteristics	Categories	*N* (%)
Age, years (*n* = 4,352)	Median, IQR	65 (53–74)
Sex (*n* = 4,351)	Male	1,830 (42)
	Female	2,521 (58)
Race (*n* = 4,130)	White	3,466 (84)
	Black	302 (7)
	Asian	223 (5)
	American Indian or Alaska Native	13 (0)
	Native Hawaiian or Other Pacific Islander	1 (0)
	Other/Multiple	85 (2)
	Declined	40 (1)
Ethnicity (*n* = 4,122)	Hispanic or Latino	264 (6)
	Non-Hispanic/Latino	3,781 (92)
	Declined	77 (2)
Language (*n* = 4,346)	English	4,321 (99)
	Spanish	11 (0)
	Other	14 (0)
Appointment type (*n* = 4,353)	Office visit	4,203 (97)
	Video visit	6 (0)
Patient type (*n* = 4,354)	Returning patient	3,859 (89)
	New patient	495 (11)
Appointment Status (*n* = 4,354)	Completed	4,203 (97)
	Canceled	138 (3)
	Left without seen	4 (0)
	No Show	9 (0)
FHS7 Response Method	Electronic patient portal (MyChart)	3,769 (87)
	In-person at clinic (EpicCare)	586 (14)
Time of Day	Morning (6am-12pm)	2,075 (48)
	Afternoon (12pm-6pm)	1,513 (35)
	Evening (6pm-12am)	467 (11)
	Night (12am-6am)	300 (7)

Since item non response can impact screening outcomes, we evaluated patterns and factors associated with responding to the adapted FHS7. In multivariable regression, questionnaire completion was lower among MyChart respondents (OR = 0.33, CI: 0.24–0.44) compared to in-clinic completion, and among Black and Asian patients compared to White patients (OR = 0.6, CI: 0.46–0.80, and OR = 0.72, CI: 0.53–0.99, respectively). Completion was positively associated with being female (OR = 1.54, CI:1.32–1.79) and non-Hispanic or Latino (OR = 1.59, CI: 1.16–2.15). These trends were largely driven by differences in responding to numeric vs. dichotomous items, with higher item non-response of numeric items (Table 4).

**Table 4:** Multivariable regression of factors associated with item non-response of the adapted family history screening tool (*N* = 3,905)

Category	Variable	Dichotomous Items			Numeric Items			All Items		
		OR	95% CI	*p*-value	OR	95% CI	p-value	OR	95% CI	*p*-value
Age	-	**1.02**	**1.01–1.03**	**< 0.001**	1.00	0.99–1.00	0.368	1.00	0.99–1.01	0.972
Sex	Male	Ref	-	-	Ref	-	-	Ref	-	-
	Female	0.83	0.65–1.06	0.14	**0.69**	**0.59–0.80**	**< 0.01**	**1.54**	**1.32–1.79**	**< 0.01**
Race	White	Ref	-	-	Ref	-	-	Ref	-	-
	Black	1.41	0.88–2.17	0.13	**1.42**	**1.06–1.89**	**0.02**	**0.60**	**0.46–0.80**	**< 0.01**
	Asian	0.97	0.52–1.69	0.94	**1.44**	**1.04–1.97**	**0.03**	**0.72**	**0.53–0.99**	**0.04**
	Other	0.56	0.14–1.55	0.34	1.23	0.75–1.98	0.40	0.76	0.47–1.24	0.25
Ethnicity	Hispanic/Latino	Ref	-	-	Ref	-	-	Ref	-	-
	Non-Hispanic/Latino	1.26	0.70–2.50	0.48	**0.61**	**0.45–0.83**	**< 0.01**	**1.59**	**1.16–2.15**	**0.03**
FHS7 Response Method	PCP-Administered	Ref	-	-	Ref	-	-	Ref	-	-
	Self-Administered	**1.99**	**1.29–3.24**	**< 0.01**	**3.45**	**2.54–4.80**	**< 0.01**	**0.33**	**0.24–0.44**	**< 0.01**
Patient Type	New Patient	Ref	-	-	Ref	-	-	Ref	-	-
	Established Patient	0.98	0.65–1.55	0.93	1.14	0.88–1.48	0.34	0.84	0.64–1.08	0.18

Statistically significant items are in bold

### The impact on fidelity

For a subset of patients with available data, we examined the validity of self-reported family history data against GC-collected family history data. Of the 3,905 patients who self-reported their family history, 339 (9%) also underwent genetic counseling for cancer risk assessment. Comparisons of self-reported and GC-collected family history data are shown in Table 5. For binary (yes/no) questions 1–6, the percentage total agreement on questions ranged from 67 to 96%, with specificities ranging from 68 to 98%. Concordance was best for male family history of breast cancer (96%) and worst for family history of breast cancer in first-degree relatives (67%). For questions with numeric answer choices [7–9], intraclass correlation coefficients indicated large within-group variability for all questions.

**Table 5:** Comparison of self-reported and genetic counselor documented family history data and estimates of validity measures among primary care patients (*n* = 339)

Items of Adapted FHS7	Self-report	GC-documented	
**Binary questions**:	**N = Yes (%)**	**N = Yes (%)**	**% Agreement**,** Concordance** (K, 95% CI)
1. Did any of your first-degree relatives (parent, children, siblings) have *breast* cancer?	177 (52)	168 (50)	67% 0.35 (0.25, 0.45)
2. Did any of your first-degree relatives (parent, children, siblings) have *ovarian* cancer?	39 (12)	19 (6)	88% 0.22 (0.06, 0.37)
3. Did any of your relatives have *bilateral breast* cancer (i.e., cancer in both breasts)? (yes/no)	52 (15)	10 (3)	85% 0.12 (0.0001, 0.24)
4. Did any man in your family have *breast* cancer?	9 (3)	6 (2)	96% 0.12 (−0.12, 0.35)
5. Did any woman in your family have both *breast and ovarian* cancer?	17 (5)	6 (2)	94% 0.15 (−0.06, 0.36)
6. Did any woman in your family have *breast* cancer before the age of 50 years?	101 (30)	108 (32)	68% 0.26 (0.15, 0.37)
**Numeric questions**:	**Mean ± SD**	**Mean ± SD**	**ICC** (95% CI)
7. How many relatives with *breast* cancer are in your family?	1.3 ± 1.5	1.6 ± 1.4	0.19 (0.09, 0.29)
8. How many relatives with *bowel* cancer (colon/rectal) are in your family? _____	0.5 ± 0.8	0.5 ± 0.9	0.21 (0.11, 0.31)
9. How many relatives with *ovarian* cancer are in your family? _____	0.2 ± 0.4	0.2 ± 0.4	0.06 (−0.05, 0.17)

## Discussion

In this study, we find that adapting FHS7 from provider report to patient report helped implementation of the guideline-recommended HBOC risk assessment tool in this primary care setting. In our adaptation, we retained the core function (i.e., effective and necessary component) - to evaluate specific family history of cancer information, while the form was adapted to fit the context in which the intervention was deployed. We found that most patients attempted to complete the questionnaire via MyChart, and 77% of patients successfully responded to all nine items of the adapted FHS7. We believe that collection of family history information primarily through MyChart before primary care appointment combined with high MyChart uptake at the clinical setting was helpful in implementing FHS7 and achieving high response rates.

We piloted the adapted FHS7 to evaluate its performance in the primary care setting. While the overall response rate was high, our analysis noted lower response rates for questions that required numeric answers compared to questions with binary responses, indicating that some response formats may require more interpretive effort for respondents, resulting in lower response. It may also indicate patients’ inability to correctly recall specific family history information, especially quantifying the number of family members with a specific degree of relatedness and types of cancer. A recent study found that a self-administered but not adapted FHS7 questionnaire had a positivity rate of 3.6% which is substantially lower than the 23.8% reported by Ashton-Prolla et al. [14]. We report a FH7 positivity rate of 36% which is closer Ashton-Prolla’s [3]. Thus, some form of adaptation of a tool intended for PCP administration may be necessary to produce comparable results when patients use the tool to self report family history. Notably, EHR data alone vastly underestimated the number of FHS7 positive patients suggesting that direct questionnaires will improve data reliability and yield more comprehensive assessment than EHR-derived data alone. Thus, while the FHS7 positivity rate derived from patient self-report data using the adapted FHS7 may not precisely estimate the true prevalence, the data collection method is more suited to implementation in clinic where relying on healthcare providers alone prevent implementation of risk-assessment altogether. Since the adapted questions asked for an exact number of relatives whereas the original asked if patients had 2 or more relatives, future adaptations should consider reverting to increase item-response rates.

For the subset of patients with both self-reported and genetic GC-collected family history data, we found generally high percentage agreement but low kappa, suggesting the need for more sample sizes for that subset analysis. Compared to GC-collected data, patients overestimated their cancer family history in self-report. Our findings are consistent with a recent report showing that patients report greater numbers of first-degree relatives with cancer when information is collected at home than at an office visit [20]. Studies also show that breast and colon cancer history is reported with more accuracy than other obscure site-specific cancers [21], first-degree history is more accurate than second-degree history [21], and men are less familiar with family history of cancer than women [22]. The overestimation of family history in our study may be a product of the selected and small sample in which analyses were performed. Its limitations notwithstanding, family history collection remains a critical component of health risk assessment, especially in hereditary cancer. The FHS7 adaptation enabled patients to report their family history information at home, before their medical appointment, thus facilitating hereditary cancer risk assessment at this primary care setting. Of note, adaptation and validation are distinct but crucial steps for ensuring appropriateness of programs or tools in specific contexts. Here we focused on adaptation and report preliminary data on validation.

Clinical settings need routine and accurate collection of family health information. While data collected by healthcare providers (PCP or GC) yield more accuracy, the data collection is less routine due to time limitations and barriers to accessing care. By implementing a patient-reported family history screening tool, followed by primary care review, our work provides a path toward implementation of guideline-recommended preventative care [4, 23, 24]. Our adaptation of an existing efficacious data collection tool reduced demand on primary care provider time. We selected an tool with strong evidence base that is recommended by USPSTF, simple to administer and evaluate, and preferred by community members [13]. A multidisciplinary team adapted FHS7 following best practices for patient-centered communication (e.g., fewer jargons, clarifying medical terminology). A Key lesson learned in the adaptation process was the importance of involvement of stakeholders, including clinical informaticians, clinic champions, genetic counselors, and behavioral scientists.

As with any screening test, tradeoffs between sensitivity and specificity require value judgements. Because follow-up procedures for family history screening (i.e., assessment by healthcare provider) are neither invasive nor expensive, a higher false positive may be permissible. Here we generated preliminary evidence for systematic implementation of hereditary cancer risk assessment in primary care and support for providers who are well- positioned to provide preventive genetic care. The results of the complete hereditary cancer screening program will be reported in a subsequent paper.

There are several study limitations. First, the patient population served at the clinic is not representative of all primary care patients, which limits generalizability of study findings. Second, the adaptation procedures could have been further enhanced by conducting cognitive interviews with primary care patients prior to pilot implementation. However, because the adaptations were based on a strong body of literature on designing patient-reported materials, we felt we could move forward without cognitive testing. Third, GC-derived FHS7 data were only available for FHS7-positive patients, thus precluding us from doing a real validity analysis that requires data from patients with both positive and negative tests. Still, the data presented provide a partial measure of concordance between self-reported family history and the gold standard. Future work should comprehensively evaluate quality and accuracy of self-reported family history information using the adapted FHS7 compared to GC-collected information, as both validation and adaptation are key to ensuring appropriateness of tools in specific contexts.

## Conclusions

In conclusion, adaptation of FHS7 from a provider-reported to patient-reported instrument was key to implementation of the hereditary cancer risk assessment program in a primary care setting. Fidelity to the core function allowed us to use the same algorithm on the self-reported data. Data also support the practice of collecting cancer family health history directly from patients for hereditary cancer risk assessment, thus making it feasible to implement hereditary cancer risk assessment at other settings with limited PCP involvement.

## Data Availability

The datasets used and/or analysed during the current study are available from the corresponding author on reasonable request.

## Abbreviations

FHS7: Family History Screening 7
PCP: Primary Care Provider
GC: Genetic Counselor
EHR: Electronic Health Record
FRAME: Framework for Reporting Adaptation and Modifications to Evidence-based Implementation Strategies
HBOC: Hereditary Breast and Ovarian Cancer
